# Cardiology—Abderhalden's Test—Leukæmia—Sciatica

**Published:** 1914-02-21

**Authors:** 


### MODERN METHODS SERIES.—III.

### Cardiology—Abderhalden's Test—Sciatica.

It has been a question of great interest whether the heart laboratory would confirm the views of the older schools in regard to strychnine and digitalis, as well as in connection with diffusible stimulants such as the ammonia preparations and ether, upon which physicians have so long relied to restore patients suffering from heart failure. Results obtained in the recently constituted cardiological department at the London Hospital show that research has fully established the usefulness of digitalis, and, further, that the cardiologists of to-day have even greater respect for this drug than the physicians whose knowledge of its action was more or less empirical. On the other hand, there are those who consider that strychnine has been proved quite valueless as a heart stimulant, and refer to cardiograms and other graphic records as evidence that this alkaloid has no demonstrable effect on either heart or blood-pressure except in poisonous doses. Similarly the old-fashioned mixtures containing ammonia and ether are said to be of little, if any, practical value as direct cardiac stimulants, although it is admitted that they may produce beneficial effects through relieving a flatulent stomach, or reflexly through that organ.

On the other hand, it has been found that the diffusible stimulants which have been proved unreliable can be well replaced by strophanthin, which is now given intravenously in urgent cases, and may be relied upon to act within half an hour. It is considered that strophanthin is much more reliable than the digitalin commonly obtainable, as the latter appears to vary in strength and efficiency.

A patient suffering from heart disease who undergoes treatment according to modern principles is watched carefully from day to day, accurate records being kept of the pulse-rate whilst at rest and after slight exertion, and a sufficient dose of digitalis (in the form of standardised preparations) given to keep the pulse steady. An endeavour is made to produce a partial heart-block through the selective action of digitalis on the auriculo-ventricular bundle of His, so that irregular stimuli from the distended auricle are not transmitted to the ventricle, which is, therefore, free to beat at its own intrinsic rhythm. In such circumstances the ventricle is not exhausted by premature or useless contraction, and beats sufficiently with obvious benefits to the whole system. Apart from this, the tonic action of digitalis on heart-muscle is freely recognised, and full advantage taken of its action as hitherto.

### Abderhalden's Test.

Dr. Abderhalden, the well-known physiologist of Halle, holds that the body automatically endeavours to protect itself against toxins associated with various diseases by the production of specific ferments. On this theory he has designed a diagnostic method which depends on the demonstration of such new substance in the blood-serum, and during the past year some experiments which tend to establish the truth of the hypothesis have been carried out at St. Bartholomew's Hospital. Mr. R. St. Leger Brockman, who is responsible for this work, has satisfied himself that the test is one of practical use in the differential diagnosis of cancer, and has recently reported his results to the *Lancet* (November 15, 1913), at the same time giving details of his method. In this, advantage is taken of the fact that the new substance found in the blood-serum of persons suffering from cancer will pass through an osmotic membrane. Serum drawn off from a suspected case is subjected to dialysation, the dialysate being subsequently tested with triketohydrindene hydrate (ninhydrin), in contact with which it gives a violet colouration when heated. In practice a small portion of carcinoma tissue is placed in a dialysing tube with the serum to be tested, the tube then being placed in sterile distilled water. The dialysing apparatus is incubated at 37 deg. C. for twelve to twenty-four hours. Mr. Brockman's conclusions, as then reported, are to the effect that there is definite reason to believe that in carcinoma blood contains a new substance which has a specific proteolytic action on cancer tissue only. It is to be noted that similar conclusions have been arrived at by other workers, so that it is not unlikely that the method will sooner or later become helpful in routine work.

### Treatment of Sciatica.

From results that have been obtained lately in the treatment of sciatica by light and static electricity it would appear that many more cases of this troublesome malady might thus be relieved were the advantages of this method of treatment more generally known to practitioners. While successes are claimed by the advocates of both light and electricity, particularly good results can be obtained by the use of them in combination. Using a very powerful lamp the painful area is subjected to its rays for the best part of half an hour; the patient is then seated on the platform of a static machine, and given wave-current for another quarter of an hour or so. Further, in some cases he is given sparking treatment from the static apparatus along the course of the sciatic nerve. It is supposed that the physical result of this combined treatment is to cause flushing of the affected part with blood and resolution of inflammatory material.

